# Relationship Between the Level of Vitamin D3 Deficiency and Successful Osseointegration: A Prospective Clinical Study

**DOI:** 10.1155/2024/9933646

**Published:** 2024-09-30

**Authors:** Ahmed Fadhel Al-Quisi, Firas A. Jamil, Auday M. AL-Anee, Salah Jassim Muhsen

**Affiliations:** ^1^ Oral and Maxillofacial Surgery Department College of Dentistry University of Baghdad, Baghdad, Iraq; ^2^ Oral and Maxillofacial Surgery Unit at Al-Kindy Teaching Hospital, Baghdad, Iraq; ^3^ Oral and Maxillofacial Surgery Unit at Al-Shaheed Gazi Al-Hariri Teaching Hospital, Medical City, Baghdad, Iraq

**Keywords:** bone density, dental implant, osseointegration, vitamin D3

## Abstract

**Purpose:** This study aimed to evaluate the influence of vitamin D3 levels on bone density, primary dental implant stability, and successful osseointegration.

**Materials and Methods:** Clinical and radiological examination with a standardized cone-beam computed tomography (CBCT) machine and laboratory investigation for serum levels of vitamin D3 were performed for all patients. Only patients in need of single or multiple straightforward dental implant surgery in either jaw with no history of systemic disease or condition that may interfere with bone healing were included in this study to receive the dental implant by the same oral and maxillofacial surgeon, which re-opened 4 months later to assess the osseointegration and to complete the prosthetic part.

**Results:** One hundred twenty-eight dental implants were inserted into 108 patients. Most of the patients in the study had insufficient vitamin D3 levels. The prognosis of dental implants regarding successful osseointegration 4 months after implant placement had a weak positive association with the insertion torque and bone mineral density and a statistically significant positive correlation with the serum vitamin D3 level.

**Conclusion:** Preoperatively, it is advisable to request the serum vitamin D3 level of the patients along with the standard clinical and radiological examination. Severe vitamin D3 deficiency could be associated with early dental implant failure despite the favorable bone density and primary dental implant stability achieved.

**Trial Registration:** ClinicalTrials.gov identifier: TCTR20200304001.

## 1. Introduction

Once the dental implant is inserted into the alveolar bone of either jaw, many factors may affect the process of osseointegration. These factors may vary from one individual to another and from one place in the oral cavity to another. One of these factors is bone density, which affects primary implant stability, predicting the outcome of dental implant treatment [1]. Besides quality, bone quantity influences primary dental implant stability, which is crucial for achieving osseointegration [2–4].

Dental implant geometry and thread design contribute to the primary dental implant stability by controlling the amount of the bone trabeculae that come into contact with the implant surface and influencing the longevity of the osseointegration and dental implant success after loading the dental implant-supported prosthesis [5, 6].

Vitamin D is a term used to describe the collection of vitamins D3 (cholecalciferol) and vitamin D2 (ergocalciferol) [7]. Vitamin D3 influences bone metabolism and remodeling by affecting phosphorus and calcium metabolism, bone matrix ossification, and bone density [8, 9]. Constant low intake of vitamin D3 can lead to vitamin deficiency, leading to reduced bone mineral density and osteoporosis [10, 11]. Vitamin D3 deficiency may also induce secondary hyperparathyroidism, leading to further bone demineralization and decreased bone density [12].

Estimation of the bone density from cone-beam computed tomography (CBCT) was not accurate because it depends on inconsistent grayscale values; on the other hand, computed tomography (CT) can give Hounsfield units (HU) values that are standardized across various CT machines which lead to more accurate determining of the tissue density [13]. A recent clinical study showed a novel approach in standardizing the grayscale values in CBCTs to effectively calculate Hounsfield units (based on grayscale values) within approximately the same variability range observed from the CT scans [14].

Different methods are used to estimate dental implant stability. Dental implant insertion torque, expressed in the values of Ncm, is considered a valid indicator of implant stability at the time of dental implant insertion. Another noninvasive method is resonance frequency analysis (Osstell), which includes implant stability measurement with a transducer (smart peg) fixed into the implant fixture and excited through a range of sound frequencies; the resulting measurement of the dental implant vibratory oscillation will be shown on the device's digital screen [15, 16].

For a better understanding and accurate assessment of the dental implants' early success, Tolstunov, in 2007, classified dental implant regions in the jaws into four different zones. The upper central and lateral incisors, canines, and first premolars were included in Zone 1 (traumatic zone). In contrast, the upper second premolar and the molars were included in Zone 2 (sinus zone). At the same time, the lower central and lateral incisors, canines, and first premolars were included in Zone 3 (interforaminal zone). In contrast, lower molars with second premolars were included in Zone 4 (ischemic zone) [17].

An animal study in 2008 showed the negative effect of vitamin D3 deficiency in the bone-to-implant integration process in the second week of healing. However, the long-term effects remain unknown [8]. Another retrospective clinical study in 2016 finds that patients with severe deficiency state may had a high early implant failure rate of 9% when compared to patients who had sufficient levels of vitamin D3 with a failure rate of 2.2% [18]. In contrast, a histomorphometric study by Schulze-Späte et al. investigated the influence of vitamin D3 and calcium supplementation on the bone remodeling of the sinus bone graft. There were no significant differences between the placebo group and the study group [19].

Despite many shreds of evidence that report deleterious effects on bony tissues with severe vitamin D3 deficiency, there is no clear relationship demonstrated in prospective clinical studies between severe vitamin D3 deficiency and early failures of osseointegration around dental implants [20]. This study aimed to evaluate the influence of vitamin D3 serum levels on bone density, primary dental implant stability, and successful osseointegration.

## 2. Materials and Methods

This prospective clinical study was conducted in the Oral and Maxillofacial Surgery Unit, Dental Teaching Hospital, according to ethical principles and in compliance with the Declaration of Helsinki and its later amendments. It was registered in the Thai Clinical Trial Registry (TCRT) on 4 March 2020 with an ID of (TCTR20200304001) and ethically approved by the local ethical committee at the College of Dentistry/University of Baghdad with project number 772,123. The sample calculation of the sample size was achieved by G-power software using a *t*-test point bi-serial model of correlation (two-tailed) and a medium effect size of 0.3, an alpha error of 0.05, and a power of 0.085. The resulting sample size was 108 patients after adding the potential dropout.

The included patients were examined clinically by an independent maxillofacial surgeon; any patient above 18 years old with sufficient bone volume and in need of single or multiple straightforward dental implant surgeries in either jaw with no history of systemic disease or condition that may interfere with bone healing were included in this study. In comparison, patients with a history of smoking, uncontrolled diabetes, immunosuppression, history of radiotherapy, and insufficient alveolar bone volume (width and height) for installation of dental implants were excluded.

The patient's jaw areas were divided into four zones, according to Tolstunov (2007) to differentiate between regions with different characteristics [17].

**Figure 1:**
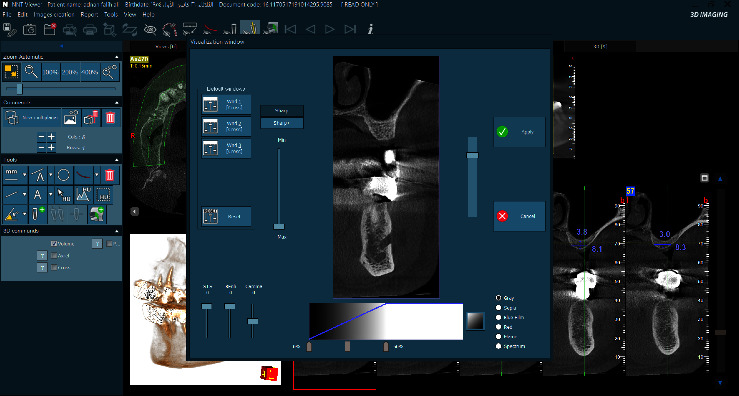
NNT viewer software front page showing the settings used in this study.

Furthermore, the CBCT scan was standardized for all patients using the NewTom GO 2D/3D/CEPH unit, with exposure setting (Eco scan) FSV 9 k.V, SSV 8 m.a. A standard exposure time of 3.7 s and field of view collimated to 10 ∗ 10 cm hires modality. The resulting DICOM files were evaluated using NNT viewer software after adjusting the sharpness to the maximum and contrast to 50%, as seen in Figure 1.

**Figure 2:**
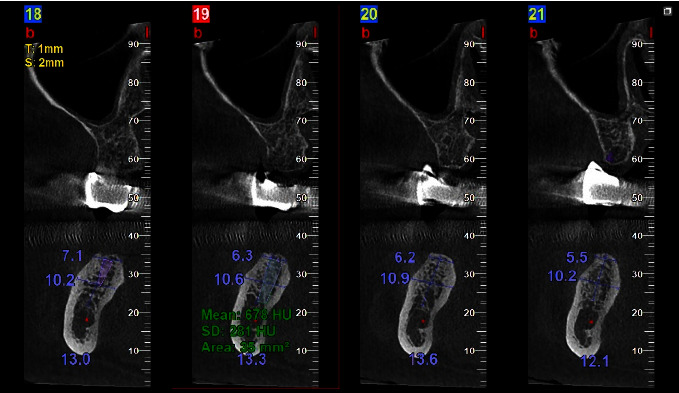
CBCT's coronal view showing a virtual dental implant with the estimated bone density inside it.

A virtual dental implant (diameter 3.8/length 11) was inserted into the planned surgical site in a 1 mm subcrestal position without including the buccal or palatal cortical plates. Tracing was made to measure the mean bone density inside that virtual dental implant (Figure 2), and then these densities were sorted according to the Misch classification [21].

Preoperatively, informed consent was obtained from all patients regarding the procedure and the vitamin D3 serum level (assessed by drowning a 2 cc of the patient's venous blood into a glass tube and sending it to the nearby specialized laboratory) and then classified into three groups of deficiency, insufficiency, and sufficiency according to their serum level measured in ng/mL units. Serum levels above 30 ng/mL were considered sufficient, while levels between 20 and 29 ng/mL were considered insufficient, and those below 20 ng/mL were deficient [22].

A standard surgical protocol for dental implant insertion was performed by the same oral and maxillofacial surgeon for all patients, which included infiltration of the local anesthetic solution into the buccal/labial and palatal/lingual soft tissues, followed by crestal incision made by blade no. 15 fixed on blade handle no. 3 over the intended dental implant site and extended around the adjacent teeth (one tooth next to the drilling site both mesially and distally). Then Haworth's periosteal elevator was used to reflect full thickness mucoperiosteal flap followed by a sequential drilling technique according to the manufacturer's instruction from the MEDENTiKA® dental implant system from (Hügelsheim, Germany) with diameters of 3.8, 4.1 mm and length of 11 mm. The insertion torque value of all dental implants was recorded using a graded torque ratchet. After 4 months, the surgical site was re-opened to insert a healing abutment and to complete the prosthetic part by the same surgeon who performed the initial surgical intervention.

The study's statistical analysis was achieved using the package for social sciences program (SPSS) version 24.0 (SPSS Inc, Chicago, IL, USA). A Shapiro–Wilk test was performed first to check the normality of the data distribution. Spearman's Rho Calculator was used to measure the strength of the association between two variables when *r* value = 1 means a strong positive correlation and when *r* = −1 means a perfect negative correlation. In contrast, the Point-Biserial Correlation Calculator was used to measure the correlation between two variables when one of the study variables is dichotomous. If the *p* value is less than 0.05, then the association between the two continuous variables is statistically significant.

## 3. Results

A total of 176 patients were initially screened for possible participation in this study; 68 patients were excluded (44 had narrow ridges, 14 had diabetes, and 10 patients were heavy smokers), and the remaining 108 patients (72 females and 36 males) with one hundred twenty-eight dental implants were included in this study. The mean age of these patients was 52.81 ± 11.14 years. Most of the patients in the study had insufficient vitamin D3 levels. In comparison, 35 patients had sufficient vitamin D, and only 27 were diagnosed with vitamin D3 deficiency (Table 1).

Only two implants failed to osseointegrate into the alveolar bone in Zone 1 of female patients with the lowest vitamin D3 level (the only patients with a vitamin D3 level below 5) (Table 2).

The greater insertion torque was recorded in the Zone 3 (mandibular incisors, canine, and first premolars) area, and it was (31.07 ± 8.00) followed by the Zone 4 (mandibular second premolars and molar) region with a mean of (30.71 ± 5.96). The mean of the insertion torque in Zone 1 (maxillary incisors, canine, and first premolars) area was (28.24 ± 6.47), and the lowest mean of the insertion torque was recorded in Zone 2 (maxillary 2^nd^ premolars and molars) with a mean of (22.77 ± 6.46). Further information regarding the number of the patients and dental implant according to the site and correlations between vitamin D3 and bone density is detailed in Table 3.

On the other hand, there was a statistically significant association between the mean insertion torque for the dental implants and the mean bone densities with *r*_*s*_ of 0.72355.

However, the prognosis of the dental implants regarding successful osseointegration after 4 months from the initial implant insertion surgery had only a weak positive association with the insertion torque and bone density and a statistically significant positive association with the serum vitamin D3 level, as shown in Table 4.

## 4. Discussion

Few studies in the literature have evaluated the impact of the low level of vitamin D3 on dental implant osseointegration; most of these studies are case reports or retrospective clinical studies with two randomized control trials designed to investigate the influence of the vitamin D3 serum level on the marginal bone loss and implant survival rate.

This prospective clinical trial is designed to investigate the influence of the serum vitamin D3 level on dental implant osseointegration 4 months after the initial surgical phase. This time frame was selected to avoid confounding factors of the prosthetic parts (load, design, and cleaning) that may affect dental implant survival in healthy, nonsmoker patients with adequate alveolar bone volume.

In addition, this study correlated the bone density obtained from a standardized CBCT machine with the serum level of vitamin D3 and dental implant insertion torque. Since successful dental implant osseointegration is the surgeon's prime goal, multiple local and systemic factors should be considered, like infection, smoking, lack of healthy and appropriate width of keratinized mucosa, uncontrolled diabetes, and poor blood supply to the surgical site [23].

The influence of the serum vitamin D3 level on dental implant osseointegration, bone density, and bone remodeling was demonstrated in many previous studies [8–10]; however, the association between the exact deficiency level of vitamin D3 and early implant failure in a human being is not yet established.

Only 35 patients in this study, which represent (32.40%) of the total sample size, showed a sufficient level of serum vitamin D3; these findings agreed with worldwide reports of the high prevalence of vitamin D3 deficiency [24–26]. Furthermore, these results coincide with the previous study investigating vitamin D3 levels in the Middle East [27]. Despite this region's sunny days, these findings are unsurprising, as people avoid direct exposure to strong sunlight in such semidesert areas.

A recent clinical study by do Vale Souza et al. showed a strong positive correlation between implant insertion torque values in N/cm and the implant stability quotients (ISQ) values generated from the Osstell device [28].

The ease of availability of the torque ratchet, as it is part of the dental implant kit, and the presence of surgical micromotors that facilitate the assessment of the implant insertion torque during implant fixture placement made it an attractive choice for measuring primary implant stability.

The greatest dental implant insertion torque was recorded in Zone 3, associated with the highest bone density obtained from grayscale values of standardized CBCTs. The lowest insertion torque was recorded in the Zone 2. This statistically significant positive correlation between dental implant insertion torque and alveolar bone density measured in CBCT goes in line with studies that concluded that increased bone density, when evaluated both histologically or clinically, had a strong significant correlation (*p* 0.0001) with insertion torque and in the same order of the jaws regions as in the current study [29, 30].

However, only a weak positive correlation was calculated between vitamin D3 at all levels and alveolar bone density at different jaw regions. As a result, the presence of good bone density in CBCT does not mean the presence of optimum serum level of vitamin D3, and this appeared with the two failed dental implants, where the optimal insertion torque and good bone density did not prevent failure of osseointegration when the serum vitamin D3 level is severely deficient. Furthermore, an animal study by Mengatto et al. in 2011 showed that bone-implant contact was significantly reduced despite normal volumetric bone growth after dental implant installation in Sprague–Dawley rats subjected to the vitamin D3 deficient protocol [31]. Three years later, a case report by Bryce and MacBeth showed that vitamin D3 deficiency is strongly related to early dental implant failure [32]. The results of these previous studies strongly support the current study's findings that showed a strong positive correlation between vitamin D3 levels and successful dental implant osseointegration.

However, a retrospective clinical study by Mangano et al. in 2016 found no clear relationship between low-level serum vitamin D 3 and early dental implant failure; the same author published another retrospective study in 2018 with the same conclusion despite the higher number of dental implants failed in the vitamin deficient group [18, 33]. Mengatto et al. results may be influenced by the number of patients with vitamin D3 deficiency in the study, constituting about (3.15%) of the total samples. In contrast to the current study, 25% of the total samples are patients with vitamin D3 deficiency. Furthermore, including patients with comorbidities (heavy smokers and severe periodontitis) in these retrospective studies may have influenced their results.

Within the study's limitations (lack of diet habit survey, serum alkaline phosphatase, serum calcium, and haemoglobin blood levels for the included patients, which may contribute to bone healing), it can be concluded that preoperative requesting of serum vitamin D3 level in conjunction with the standard clinical and radiological examination is advisable. Severe vitamin D3 deficiency could be associated with early dental implant failure despite the favorable bone density and primary dental implant stability achieved.

## Figures and Tables

**Table 1:** Demographic distribution of the patients and dental implants according to the level of the serum vitamin D3.

	**No. of patients**	**Implant sites and number**	**Total number of implants**	**Vitamin D3 level**	**Vitamin D3 (ng/dL) (mean ± SD)**	**Bone density (grayscale) (mean ± SD)**	**r**_**s**_
Zone 1	39 (11 male and 27 female)	Central incisor (11)	41	Deficiency	8.83 ± 2.44	493.90 ± 226.11	0.37
		Lateral incisors (9)		Insufficiency	22.02 ± 5.47	495.29 ± 219.98	0.50
		Canines (7)		Sufficiency	39.62 ± 15.00	425.21 ± 266.71	0.20
		1^st^ premolars (14)					

Zone 2	16 (4 male and 12 female)	2^nd^ premolars (0)	18	Deficiency	8.46 ± 1.60	294.66 ± 233.38	0.18
		1^st^ molars (12)		Site	16.66 ± 0.81	367.00 ± 265.97	0.31
		2^nd^ molars (6)		Sufficiency	38.00 ± 9.48	310.50 ± 119.95	0.08

Zone 3	16 (3 male and 13 female)	Central incisor (1)	19	Deficiency	9.50 ± 0.57	821.50 ± 311.10	0.44
		Lateral incisors (8)		Insufficiency	20.00 ± 3.55	825.00 ± 109.76	0.80
		Canines (3)		Sufficiency	47.12 ± 9.32	849.12 ± 322.20	0.46
		1^st^ premolars (7)					

Zone 4	38 (14 male and 24 female)	2^nd^ premolars (0)	42	Deficiency	9.09 ± 1.81	531.80 ± 200.90	0.59
		1^st^ molars (25)		Insufficiency	19.55 ± 4.35	829.44 ± 354.00	0.02
		2^nd^ molars (17)		Sufficiency	47.12 ± 9.32	849.12 ± 322.20	0.24

**Table 2:** Detailed information regarding the failed dental implants.

**No.**	**Sex**	**Age (years)**	**Serum vitamin D3 level (mg/dL)**	**Site of the dental implant**	**Preoperative bone density (grayscale)**	**Size of the dental implant (mm)**	**Insertion torque (ng/dL)**	**Time of failure**
1	Female	28	4	Upper right 1^st^ premolar	721	3.8 ∗ 11	35	After 4 months from the primary surgical phase at the time of healing abutment placement
2	Female	45	3	Upper left lateral incisor	877	3.8 ∗ 11	35	The dental implant extruded into the oral cavity 2 months after the primary surgical phase.

**Table 3:** Correlations between the mean level of vitamin D3 and mean bone density according to the site of the implant placements.

**Groups**	**No. of the patients (mean ± SD)**	**No. of the implants (mean ± SD)**	**No. of the failed implants**
Deficiency	27 (25%) 9 males and 18 females	29 (22.65%)	2
Insufficiency	46 (42.59%) 12 males and 34 females	55 (42.96%)	0
Sufficiency	35 (32.40%) 15 males and 20 females	44 (34.37%)	0

*Note:* Spearman's Rho Calculator.

**Table 4:** The correlations between early dental implant successful osseointegration and mean of the bone density, insertion torque, and vitamin D level.

**Correlations**	**Bone density in CBCT grayscale**	**Insertion torque value NCM**	**Vitamin D3 level (ng/dL)**
Early dental implant successful osseointegration (*p* value)	0.211	0.193	0.045^∗^

*Note:* Point-Biserial Correlation Calculator.

^*∗*^ *p* value  ≤ 0.05.

## Data Availability

The data supporting the study's findings are available from the corresponding author upon reasonable request.
